# Association between Neuroticism and Premenstrual Affective/Psychological Symptomatology

**DOI:** 10.3390/psychiatryint3010005

**Published:** 2022-01-26

**Authors:** Ajna Hamidovic, Nhan Dang, Dina Khalil, Jiehuan Sun

**Affiliations:** 1 Department of Pharmacy, University of Illinois at Chicago, 833 S. Wood St., Chicago, IL 60612, USA; 2 Department of Public Health, University of Illinois at Chicago, 1603 W. Taylor St., Chicago, IL 60612, USA; 3 Department of Public Health, Benedictine University, 5700 College Rd., Lisle, IL 60532, USA

**Keywords:** neuroticism, premenstrual dysphoric disorder, premenstrual syndrome, difficulty concentrating, low interest

## Abstract

Neuroticism and premenstrual conditions share pleiotropic loci and are strongly associated. It is presently not known which DSM-5 symptoms of premenstrual syndrome/premenstrual mood disorder are associated with neuroticism. We enrolled 45 study participants to provide prospective daily ratings of affective (“depression”, “anxiety, “anger”, “mood swings”) and psychological (“low interest”, “feeling overwhelmed”, and “difficulty concentrating”) symptoms across two-three menstrual cycles (128 total cycles). Generalized additive modeling (gam function in R) was implemented to model the relationships between neuroticism and the premenstrual increase in symptomatology. Significance level was adjusted using the False Discovery Rate method and models were adjusted for current age and age of menarche. Results of the association analysis revealed that “low interest” (*p* ≤ 0.05) and “difficulty concentrating” (*p* ≤ 0.001) were significantly associated with neuroticism. None of the remaining symptoms reached statistical significance. The late luteal phase of the menstrual cycle is characterized by complex symptomatology, reflecting a physiological milieu of numerous biological processes. By identifying co-expression between neuroticism and specific premenstrual symptomatology, the present study improves our understanding of the premenstrual conditions and provides a platform for individualized treatment developments.

## 1. Introduction

Given its strong negative emotion basis, neuroticism is considered by some as the single most important factor associated with many forms of psychopathology and behavioral health [1] with important consequences on health and longevity [2,3]. The trait significantly correlates with specific demographic and physiologic characteristics. For example, neuroticism scores tend to be somewhat higher among individuals of lower socioeconomic status [4] and mean neuroticism scores of females are significantly higher than those of males [5]. Moreover, mean neuroticism scores peak in late adolescence and moderately decline in adulthood [6–8]. Hence, menarche seems to be a particularly important temporal event, suggestive of a mediatory role of sex hormones on the expression of neuroticism.

This hypothesis is further supported by numerous studies showing an association between premenstrual states/conditions, considered to have an underlying hormonal causality [9], and neuroticism. In an early landmark study, women with high neuroticism were found to be more depressed, irritable, hypersensitive to various stimuli and fatigued during the late luteal (i.e., premenstrual) phase relative to the remaining phases of the menstrual cycle [10]. Later studies found that women with premenstrual syndrome (PMS) [11,12] and premenstrual dysphoric disorder (PMDD) [12–14] have higher neuroticism scores compared to healthy controls. Furthermore, PMS/PMDD and neuroticism share pleotropic loci. Investigators of a twin study conducted in Australia examined additive genetic influence on PMS and found a 0.62 genetic correlation between the syndrome and neuroticism scores [15]. To evaluate whether any of the heritable susceptibility to PMDD resides in heritable personality traits, Miller et al. [16] evaluated estrogen-receptor alpha (ESR-1), showing a diagnosis x genotype interaction, with presence of the minor allele of 4 single nucleotide polymorphisms (SNPs), confined to a single locus, in patients, but not healthy controls, associated with high neuroticism.

Presently, PMS/PMDD assessment in The Diagnostic and Statistical Manual of Mental Disorders 5 (DSM-5) includes an evaluation of 11 core symptoms: (1) mood swings, (2) irritability or anger, (3) depressed mood, (4) anxiety, (5) decreased interest in usual activities, (6) subjective difficulty in concentration, (7) lethargy, (8) change in appetite, overeating, or specific food cravings, (9) hypersomnia or insomnia, (10) a sense of being overwhelmed or out of control, and (11) physical symptoms such as breast tenderness or swelling, joint or muscle pain, a sensation of “bloating,” or weight gain. Items 1 through 4 are considered “affective”, while items 5, 6, and 10, are considered to be “psychological” in nature [17]. Diagnosis of PMDD, according to DSM-5, requires presence of at least one affective symptom to reach the total of 5 (out of the 11 possible) required symptoms, which must be present in most cycles from the past year and confirmed in a prospective manner for at least 2 menstrual cycles. In addition, the symptoms must be associated with clinically significant distress or interference with work, school, usual social activities, or relationship with others. Premenstrual syndrome requires the presence of 1–4 symptoms, without the requirement that one must be affective in nature. Of note, the process of establishing diagnostic criteria for PMS/PMDD has been controversial [18]. It is estimated that approximately 13–18% of women of reproductive age have premenstrual symptoms severe enough to induce impairment and distress, though the number of symptoms may not meet the five symptoms on the PMDD DSM-5 list. Moreover, the DSM-5 does not provide guidance regarding the magnitude of increase in symptomatology required to meet the diagnosis and there is no consensus on how symptom severity should be assessed [18]. Prevalence rates of both PMDD and premenstrual syndrome (PMS) can change dramatically, depending on the method of measuring symptom change [18–20]; both PMDD and PMS can be associated with functional impairment [21].

Majority of studies evaluating personality traits in PMS/PMDD relied upon retrospective recall, which conflicts with the current diagnostic standard. For example, Erenoğlu et al. [22] enrolled study participants and assigned the PMS status based on retrospective recall, finding negative correlations between Premenstrual Syndrome Scale (PMSS) scores and several Quick Big Five Personality Test (QBFPT) measures (extraversion, conscientiousness, neuroticism) and a positive correlation between PMSS scores and openness. As neuroticism refers to a predisposition toward experiencing negative affect (e.g., irritability, anxiety) and associated cognitive and behavioral characteristics [23], the negative correlation found in the study by Erenoğlu et al. [22] was the opposite of what would be expected. Indeed, a positive correlation was identified in a prospective study by Ross and colleagues [24], in which study participants completed a Menstrual Distress Questionnaire for 70 days. The researchers found that neuroticism accounted for a significant amount of variation in premenstrual negative affect, measured by the 34-symptom Moss Menstrual Distress Questionnaire.

Personality and PMS/PMDD have shared underlying neurobiological systems [25] and personality can shape how women perceive a given situation, including experiences of PMS/PMDD pathophysiology [26] and recruitment of coping mechanisms [27]. Despite the strong association between PMS/PMDD and neuroticism, it is presently not known which specific DSM-5 symptoms of PMS/PMDD are associated with neuroticism. This understanding has two important implications. First, it identifies neuroticism-based symptom expression in PMS/PMDD, thereby reflecting shared biological mechanisms and providing a greater understanding of PMS/PMDD etiology. This, in turn, expands opportunities for development of future fine-tuned diagnostic and treatment approaches. Second, though still in need of solid evidence and provided that neuroticism marks PMDD [12,28], the trait can be used to estimate the validity of present DSM-5 criteria [28]. In the present study, we implemented a data science approach, which blends aspects of statistical methods, computer science, and machine learning to provide data-driven, efficient exploration of the relationships between variables and optimized prediction of outcomes [29]. We hypothesized that two affective symptoms—irritability/anger and anxiety (DSM items #2 and #4), but not the remaining affective symptoms or psychological PMS/PMDD symptoms—would be associated with neuroticism.

## 2. Methods

### 2.1. Study Design

Premenstrual Hormonal and Affective State Evaluation (PHASE) is a single-cohort longitudinal design study with a nested human laboratory between subject experiment. The study enrolls women with regular menstrual cycles to chart their symptoms using the Daily Record of Severity of Problems (DRSP) [30], menstruation timing and ovulation during three menstrual cycles. In the third menstrual cycle, in addition to DRSP collection and LH surge testing, study participants complete: (1) blood and salivary sample collection at 8 different times of the menstrual cycle, and (2) psychosocial stress testing in the late luteal phase. Knowledge gained from PHASE is expected to increase our understanding of menstrual cycle physiology and its dysregulated states. PHASE is a registered clinicaltrials.gov study (NCT03862469).

### 2.2. Study Sample

Women between the ages of 18 and 35, with regular menstrual cycles lasting 21 to 35 days [31–34], were recruited from the general population using flyers, word-of-mouth referrals and electronic media (Facebook, Instagram, and Craigslist).

Study participants first completed an online survey, following which they were scheduled to complete an in-person screening. Before any collection of data, study participants signed a consent form, approved by the University of Illinois Human Research Protection Office. Study exclusion criteria were: (a) lifetime DSM-5 Axis I disorder, except anxiety and depression (based on the Structured Clinical Interview for DSM Disorders (SCID)), (b) current (i.e., within the past 12 months) DSM-5 major depressive disorder or an anxiety disorder (based on SCID), (c) positive urine drug screen test, (d) breath alcohol concentration > 0.00%, (e) Alcohol Use Disorders Identification Test (AUDIT) score > 7, (e) self-reported smoker or carbon monoxide concentration ≥ 6 ppm, (f) self-reported irregular menstrual cycle, (g) current pregnancy (urine test-verified) or lactation, or a plan to become pregnant, (h) moderate or high suicide risk, (i) Shipley IQ (vocabulary standard score) < 80, (j) prescription medications, and (k) hormonal contraception.

Race was self-reported by study participants. Reporting race and ethnicity in this study was mandated by the US National Institutes of Health (NIH), consistent with the Inclusion of Women, Minorities, and Children policy. Individuals participating in the poststudy survey were categorized as American Indian or Alaska Native, Asian, Black or African American, Hispanic or Latino, Native Hawaiian or Other Pacific Islander, or White based on the NIH Policy on Reporting Race and Ethnicity Data.

At the screening visit (following eligibility confirmation), study participants completed the Zuckerman–Kuhlman Personality Questionnaire (ZKPQ, Short Version [35]), State-Trait Anxiety Inventory (Y-2) (STAI Y-2 [36]), Becks Depression Inventory (BDI [37]) and Social Adaptation Self-evaluation Scale (SASS [38]. STAI Y-2, BDI and SASS were used to validate our screening procedure, which excluded study participants with DSM-5 Axis I disorders.

Once enrolled and begun their subsequent menstrual cycle, study participants completed DRSP (next section) in REDCap between 7 P.M. and midnight each day for the next three menstrual cycles.

### 2.3. Study Measures

The Daily Record of Severity of Problems (DRSP) [30] is a validated scale which measures symptoms of PMS/PMDD. Each of the 24 symptoms is rated on a scale of 1 (not at all) to 6 (extreme). The relationship between the 24 DRSP symptoms and the 11 PMDD domains is specified in Table A1. In the present analysis, we evaluated affective and psychological, but not physical, functional, or behavioral symptoms of the questionnaire. This decision was based on the evidence that personality traits are linked to regional differences in brain structure [39–41] and function [42–46]. Given the preliminary nature of the present study, we selected affective and psychological symptoms because of their hypothesized closer brain structural and functional proximity to personality traits compared to, for example, physical symptoms of PMS/PMDD. The affective and psychological DSM-5 domains and symptoms, as well as their relation to DRSP questions, evaluated in the present study are listed in Table 1.

Neuroticism was measured using The Zuckerman–Kuhlman Personality Questionnaire (ZKPQ-50-CC) [35] through a self-report questionnaire consisting of 10 true–false statements. The total is calculated by summing up the total number of true statements (each corresponding to 1 point), with 0 reflecting least and 10 reflecting most neuroticism-anxiety. We analyzed neuroticism given the strong association between PMS/PMDD and neuroticism, as discussed in the introduction section.

The Beck Depression Inventory (BDI) [37] is a 21-item, self-report rating inventory that measures characteristic attitudes and symptoms of depression. Internal consistency for the BDI ranges from 0.73 to 0.92 with a mean of 0.86. [47]. The BDI demonstrates high internal consistency, with alpha coefficients of 0.86 and 0.81 for psychiatric and non-psychiatric populations respectively [48].

The Social Adaptation Self-evaluation Scale (SASS) [38] is a 21-item questionnaire exploring patient motivation and behavior. Each item is scored from 0 to 3 corresponding to no, minimal, medium, and maximum social adjustment. The score range for the SASS total score is, therefore, from 0 to 60. Higher scores signify greater adaptation.

The STAI (Form Y) version [36] contains 20 items which measure state anxiety, and another 20 items which measure trait anxiety. It has been used widely and extensively in research and clinical settings. In the present study, we analyzed the T-Anxiety scale (STAI Form Y-2), which consists of 20 items (item 21 to item 40), measuring how the respondent “generally” feels. The items assess frequency of feeling in general (1 = almost never, 2 = sometimes, 3 = often, and 4 = almost always). Higher scores reflect greater anxiety.

### 2.4. Data Analysis

For each DRSP symptom, we calculated the degree to which a symptom demonstrated an elevation in days −6 to −1 (“pre-menstruum”) from the start of the cycle relative to days +5 to +10 (“post-menstruum”). This was done for each woman for all available cycles. We subtracted the average post-menstruum score from the average pre-menstruum score and divided this score by participant-specific variance for each symptom. This essentially yielded an effect size for each woman and for each symptom [49].

We next compared neuroticism-anxiety according to diagnosis. Of note, DSM-5 diagnosis is determined as a constellation of affective, psychological, physical, behavioral, and functional symptoms (Table A1) with a “yes” or “no” determination for presence of each symptom. The effect size greater than or equal to 1 reflects presence of a symptom [49] and we applied DSM-5 diagnostic criteria, as described in the “Introduction”. We performed the Kruskal–Wallis rank sum test, with the total neuroticism-anxiety score as the outcome and group (PMDD vs. PMS vs. healthy) as the predictor. We applied Wilcoxon rank sum tests to make pairwise comparisons between group levels with Benjamini–Hochberg corrections for multiple testing.

Our main analysis was to test associations between affective/psychological premenstrual symptoms and neuroticism. Hence, we treated affective and psychological symptoms as *continuous* variables, reflected as the effect size. The ZKPQ neuroticism-anxiety is measured on a 0–10 scale. We employed 7 separate generalized additive models [29] to study the relationship between neuroticism-anxiety score and each of the 7 affective and psychological symptoms with the total neuroticism score as the predictor and each symptom as the outcome. We adopted “gam” function in the mgcv package in R for fitting the additive models. Specifically, we utilized a spline function for the predictor with maximum degrees of freedom being 6; the best effective degrees of freedom was chosen using generalized cross validation criterion. We first constructed unadjusted models, following which we adjusted the models for current age and current age plus age of menarche based on the literature demonstrating the relationship between duration of ovulatory cycles and premenstrual symptomatology [50]. We used the False Discovery Rate (FDR) method to correct for multiple testing with a *p* value less than or equal to 0.05 considered significant.

## 3. Results

### 3.1. Study Participants

The present study involved 45 participants, approximately 26 years old on average (25.77 ± 4.88 (mean ± SD)). The participant sample was mostly White (33.33%) and Asian (37.78%). For ethnicity, 80% reported non-Hispanic. Approximately half of the participants were currently students, with majority of participants (>90%) reporting the single, never married status and approximately half reporting yearly income less than $20,000. This sample of study participants was on average approximately between normal and overweight BMI status (24.51 (4.45) (mean ± SD)), with the average age of menarche being 12 years old. Please see Table 2 for additional participant characteristic details.

Of the 45 study participants, 7 met the diagnosis for PMDD, 21 met the diagnosis for PMS, and 17 participants were healthy. The diagnosic groups did not differ on the study demographic characteristics (Table A2). The analysis of neuroticism revealed that the PMDD group had higher neuroticism scores relative to the PMS and healthy control (*p*-adj = 0.028 for both). There was no difference in neuroticism scores between the PMS and healthy control groups. Our methodology of screening out participants with depression or anxiety seemed appropriate according to the results of STAI Y-2, BDI and SASS analyses, which showed normal score means and ranges (Table 2).

### 3.2. Relationship between Neuroticism-Anxiety and Premenstrual Symptomatology

**Figure 1.**
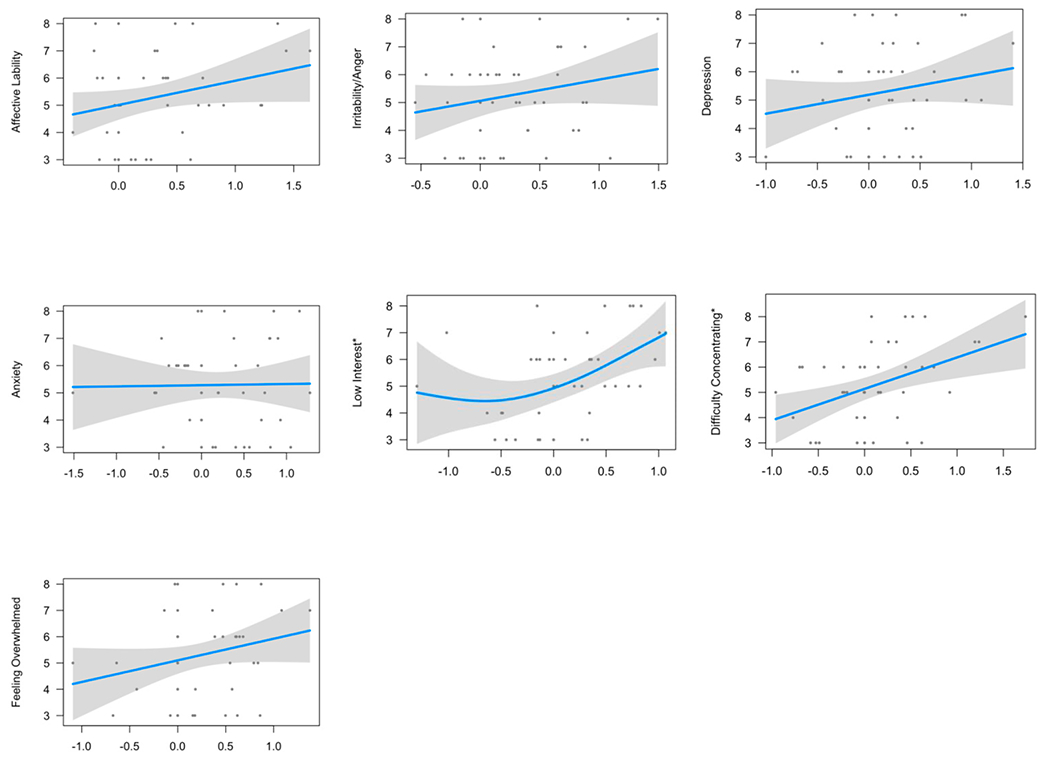
Graphical presentation of Generalized Additive Modeling (GAM) for the Neuroticism-Anxiety Personality Trait in Relation to Premenstrual Symptoms. Psychological symptoms “low interest” and “difficulty concentrating” were significantly associated with neuroticism.

Of the seven premenstrual symptoms, all except low interest displayed a linear relationship as indicated by estimated degrees of freedom (edf) of 1 (Table 3). Two symptoms (low interest and difficulty concentrating) passed the FDR-adjusted significance level. In the unadjusted models, their significance levels were 0.0392 and 0.0259, respectively. In the fully adjusted models, their significance levels were 0.016275 and 0.0003339, respectively. The highest deviance explained was for difficulty concentrating (48.9%, fully adjusted model), followed by low interest (44.1%, fully adjusted model). Figure 1 displays graphical results of the present analysis.

All models in the present study fully converged. The diagnosis of individual models indicated that the smooths had the sufficient number of basis functions (k value) to capture relationships in the data; that is, the *p* values were non-significant.

## 4. Discussion

Results of the present study show that two premenstrual psychological symptoms—low interest and difficulty concentrating—are positively associated with the neuroticism personality trait. Contrary to our hypothesis, irritability/anger and anxiety did not demonstrate a significant association. The remaining affective symptomatology was also not associated with the trait despite the affective symptom requirement in DSM-5 for PMDD diagnosis. Hence, the strong relationship between neuroticism and PMDD does not appear to be mediated by, according to DSM-5, necessary affective symptomatology.

Using stringent, prospective methodology to measure symptomatology, and a validated method to assess personality, the present study highlights co-expression of neuroticism with premenstrual decrease in interest and difficulty concentrating, which are psychological symptoms of PMDD. Our findings do not imply that neuroticism should be considered in the diagnosis criteria. Rather, the study identifies neuroticism-based symptom expression in PMS/PMDD, thereby reflecting shared biological mechanisms and expanding opportunities for development of future clinical approaches.

DSM-5 and research findings [21] emphasize high prevalence of impairment in PMS/PMDD and emphasize impairment as a metric for determining clinical significance of premenstrual symptoms. Schmalenberger et al. [17] sought to clarify symptoms that best predict three types of premenstrual impairment types (occupational, relational and recreational). The group found that affective, psychological, and physical symptoms differ in their associations with impairment. Psychological symptoms of PMS/PMDD predicted all three types of functional impairment, while affective symptoms predicted recreational and relational, but not occupational, impairment. Hence, psychological symptoms modulate functionality in a more comprehensive fashion than affective symptoms, and, as in the present study, these results highlight the importance of considering psychological symptoms in PMS/PMDD.

Anhedonia is a critical symptom of mood disorders. It predominantly manifests emotionally as a lack of feeling pleasure, as well as reduced motivation and drive on the behavioral level [51]. It is a significant predictor of psychosocial functioning [52]. In an attempt to identify the mechanisms underlying the varied symptoms associated with depression, Liao et al. [53] assessed two dimensions of Major Depressive Disorder (MDD)—anxiety and anhedonia—in relation to neuroticism, finding significant associations between neuroticism and each symptom. A critical difference between MDD and PMDD is the temporal nature of symptom expression in PMDD, as the transition between affective states, or the “switch”, in PMDD is confined to the late luteal phase of menstrual cycle, presenting a clue to its hormonal causality [9]. Results of our study, therefore, suggest that the relationship between low drive/interest and neuroticism appears to have a hormonal basis, while the relationship between anxiety and neuroticism does not, as MDD is not considered a disorder caused by shifting sex hormones (or their metabolites). Our findings, however, will need to be replicated in a study involving a larger sample size.

In addition to finding a significant relationship between low interest and the neuroticism trait, our study also revealed a significant association between premenstrual difficulty in concentrating and neuroticism. Indeed, several cognitive deficits are associated with high levels of neuroticism, including cognitive decline [54], inefficient cognitive performance [55], and increased risk of Alzheimer’s disease [56,57]. Inefficient cognitive processing in neuroticism was found to be, in part, due to elevated “mental noise” caused by preoccupations with intrusive thoughts and distress [55,58]. We show that women of reproductive age, who experience self-reported cognitive difficulties specifically in the late luteal phase of the menstrual cycle, also score high on neuroticism. A study evaluating interactions between neuroticism, shifting levels of hormones across the menstrual cycle and cognitive control is a critical future research area, with significant clinical implications.

A limitation of our study is the sample size of 45 participants. Although our analytic approach appropriately corrected for multiple comparisons, the present analysis ideally should be replicated in a larger sample size. We were not able to evaluate all DRSP items corresponding to a particular affective symptom (Table A1). For example, affective lability could be operationalized as mood swings, feeling suddenly sad or tearful, or increased sensitivity to rejection, which represent different DRSP items. To limit the number of comparisons, while being consistent in our approach, we always selected the first item on the list of affective symptoms. For example, for affective lability, we analyzed only mood swings, but not increased sensitivity to rejection. Moreover, DSM-5 specifies that premenstrual symptom increase must be “marked”, without defining what this means clinically. We applied the analytical method of calculating effect size (i.e., symptom increase) from Hartlage et al. [49], which specifies that a 100% increase in premenstrual symptomatology is clinically relevant. However, this method does not necessitate that study participants were marking their symptoms as very severe (for example, 5 or 6 on the DRSP Likert scale). A strength of our study is the prospective nature of symptom collection and an adjustment for follicular phase symptomatology, which isolates symptom exacerbation specifically in the late luteal phase of the menstrual cycle. Moreover, we measured symptoms from days −6 to −1 from the start of the subsequent menstrual cycles, which removes a possible reporting bias due to knowledge of the actual start of the period (i.e., vaginal bleeding) if the symptom measurement was, for example, from days −5 to +1, or −4 to +2. Our analysis both adjusted for multiple comparisons and important covariates.

Clarifying associations between premenstrual symptoms and neuroticism can improve the use of novel therapeutics. The present study improves our understanding of PMS/PMDD etiology, as it identified co-expression of neuroticism and hormonally-mediated exacerbation of psychological symptoms (low interest and difficulty concentrating). This can inform drug development and non-pharmacologic treatment approaches, thereby improving care for patients suffering from debilitating premenstrual symptomatology.

## Figures and Tables

**Table 1.** Domain type and symptoms of PMS/PMDD as specified in DSM-5 in relation to DRSP Questions.

Domain	DSM-5 Symptoms	DRSP Question
Affective	Marked affective lability (e.g., mood swings; feeling suddenly sad or tearful, or increased sensitivity to rejection)	DRSP 5. Had mood swings (e.g., suddenly felt sad or tearful)
	Marked irritability or anger or increased interpersonal conflicts	DRSP 7. Felt angry, irritable
	Marked depressed mood, feelings of hopelessness, or self-deprecating thoughts	DRSP 1. Felt depressed, sad, ‘down’ or blue
	Marked anxiety, tension, and/or feelings of being keyed up or on edge	DRSP 4. Felt anxious, ‘keyed up’, or ‘on edge’
Psychological	Decreased interest in usual activities (e.g., work, school, friends, hobbies)	DRSP 9. Had less interest in usual activities (e.g., work, school, friends, hobbies)
	Subjective difficulty in concentration	DRSP 10. Had difficulty concentrating
	A sense of being overwhelmed or out of control	DRSP 16. Felt overwhelmed, that I couldn’t cope

**Table 2.** Demographic, Anthropomorphic and Psychological Characteristics of Study Participants.

Variable	Mean (SD)	Percent
AGE	25.77 (4.88)	
RACE		
White		33.33
Black or African American		17.78
American Indian/Alaska Native		0.00
Asian		37.78
Native Hawaiian or Other Pacific Islander		0.00
More than one race		4.44
Unknown/do not want to specify		6.67
ETHNICITY		
Hispanic		15.55
Non-Hispanic		80.00
Do not know/Do not want to specify		4.44
STUDENT STATUS		
Yes		42.22
No		57.78
MARITAL STATUS		
Single, never married		91.11
Married		8.89
INCOME		
Less than $20,000		55.56
$20,000-$34,999		13.33
$35,000-$49,999		11.11
$50,000-$74,999		15.56
$75,000 or more		4.44
AGE OF MENACHE	12.03 (1.11)	
BMI	24.51 (4.45)	
BECKS DEPRESSION INVENTORY (BDI)	4.27 (5.14)	
SOCIAL ADAPTATION SELF EVALUATION SCALE (SASS)	46.70 (6.19)	
STATE TRAIT ANXIETY INVENTORY (Y-2)	36.37 (7.85)	

**Table 3.** Results of General Additive Modeling (GAM) for the Neuroticism-Anxiety Personality Trait in Relation to Premenstrual Symptoms.

Premenstrual Symptom	Unadjusted Model				Model Adjusted for Age				Model Adjusted for Age and Age of Menarche			
	Edf	Deviance Explained	F	*p*-Adjusted *	Edf	Deviance Explained	F	*p*-Adjusted *	Edf	Deviance Explained	F	*p*-Adjusted *
Affective Lability	1	7.24%	3.355	0.1722	1	10.7%	3.742	0.1393	1	21%	5.081	0.07536667
Irritability/Anger	1	4.64%	2.092	0.217	1	7.9%	2.352	0.166833	1	19.2%	4.399	0.079275
Depression	1	3.92%	1.754	0.224	1	7.65%	2.232	0.166833	1	14.2%	2.546	0.1708
Anxiety	1	0.0232%	0.921	0.921	1	2.91%	0.073	0.789	1	7.95%	0.55	0.465
Low Interest	2.044	23.4%	4.351	0.0392 *	1.718	24.1%	4.969	0.034195 *	2.277	44.1%	5.358	0.016275 *
Difficulty Concentrating	1	17.9%	9.394	0.0259 *	1	24.4%	12.06	0.00826 *	1	48.9%	22.62	0.0003339 *
Feeling Overwhelmed	1	5.89%	2.693	0.189	1	8.07%	2.437	0.166833	1	12.9%	2.099	0.1855

* *p* values adjusted for multiple comparisons using false discovery rate (FDR).

## Appendix A

**Table A1.** Domain type and symptoms of PMDD as specified in DSM-5.

DSM-5 Symptoms	DRSP Question	Inclusion in Present Analysis
1. Marked affective lability (e.g., mood swings, feeling suddenly sad or tearful, or increased sensitivity to rejection *^,#^	5. Had mood swings (e.g., suddenly felt sad or tearful) DRSP 6. Was more sensitive to rejection or my feelings were easily hurt	Included as affective symptom
2. Marked irritability or anger or increased interpersonal conflicts *^,#^	7. Felt angry, irritable 8. Had conflicts or problems with people	Included as affective symptom
3. Marked depressed mood, feelings of hopelessness, or self-deprecating thoughts *^,#^	1. Felt depressed, sad, down, or blue 2. Felt hopeless 3. Felt worthless or guilty	Included as affective symptom
4. Marked anxiety, tension, and/or feelings of being keyed up or on edge *^,#^	4. Felt anxious, tense, keyed up, or on edge	Included as affective symptom
5. Decreased interest in usual activities (e.g., work, school, friends, hobbies)	9. Had less interest in usual activities (e.g., work, school, friends, hobbies)	Included as psychological symptom
6. Subjective difficulty in concentration	10. Had difficulty concentrating	Included as psychological symptom
7. A sense of being overwhelmed or out of control ^#^	16. Felt overwhelmed or that I could not cope 17. Felt out of control	Included as psychological symptom
8. Marked change in appetite; overeating; or specific food cravings	12. Had increased appetite or overate 13. Had cravings for specific foods	Not included—behavioral symptom
9. Hypersomnia or insomnia	14. Slept more, took naps, found it hard to get up when intended 15. Had trouble getting to sleep or staying asleep	Not included—behavioral symptom
10. Lethargy, easy fatigability, or marked lack of energy	11. Felt lethargic, tired, fatigued, or had a lack of energy	Not included—physical symptom
11. One physical symptom (for example, breast tenderness)	18. Had breast tenderness 19. Had breast swelling, felt bloated, or had weight gain 20. Had headache 21. Had joint or muscle pain	Not included—physical symptom

* At least one symptom from items 1 to 4 in column 1 (DSM-5 Symptoms) be present for PMDD diagnosis.

# Only first symptom inside the parentheses was included in the analysis.

**Table A2.** Demographic characteristics according to diagnosis.

Demographic Variable	Category	Diagnostic Category			*p* Value
		PMDD (*n* = 7)	PMS (*n* = 21)	Healthy (*n* = 17)	
Age		25.1 (3.89)	25 (5.34)	27 (4.68)	0.27
Race	White	0.07	0.13	0.13	0.77
	Black or African American	0.02	0.07	0.09	
	American Indian or Alaska Native	0	0	0	
	Asian	0.04	0.22	0.11	
	Native Hawaiian or Other Pacific Islander	0	0	0	
	More than one race	0.02	0	0.02	
	Unknown/Do not want to specify	0	0.04	0.02	
Ethnicity	Hispanic	0.02	0.07	0.07	0.52
	Non-Hispanic	0.11	0.40	0.29	
	Unknown/Do not want to specify	0.02	0	0.02	
Student Status	Yes	0.09	0.20	0.13	0.61
	No	0.07	0.27	0.24	
Marital Status	Single	0.16	0.42	0.33	0.99
	Married	0	0.04	0.04	
	Divorced	0	0	0	
	Widowed	0	0	0	
Income	Less than $20,000	0.09	0.33	0.13	0.08
	$20,000-$34,999	0.02	0	0.11	
	$35,000-$49,999	0.02	0.04	0.04	
	$50,000-$74,999	0.02	0.04	0.09	
	75,000 or more	0	0.04	0	
Age of Menarche		12.4 (1.14)	12.1 (0.93)	11.7 (1.41)	0.21
BMI		24.0 (3.25)	24.9 (5.45)	24.3 (3.73)	0.99
